# Vulnerable Narcissism and Emotion Dysregulation as Mediators in the Link between Childhood Emotional Abuse and Binge Watching

**DOI:** 10.3390/ejihpe14100173

**Published:** 2024-09-24

**Authors:** Valeria Verrastro, Danilo Calaresi, Fiorenza Giordano, Valeria Saladino

**Affiliations:** 1 Department of Health Sciences, Magna Graecia University, 88100 Catanzaro, Italy; valeriaverrastro@unicz.it (V.V.); v.saladino@unicz.it (V.S.); 2 Department of Human Sciences, Society and Health, University of Cassino and Southern Lazio, 03043 Cassino, Italy; fiorenza.giordano@unicas.it

**Keywords:** childhood emotional abuse, vulnerable narcissism, emotion dysregulation, binge watching, emerging adults

## Abstract

Individuals who have faced childhood emotional abuse (CEA) may develop vulnerable narcissistic tendencies and experience difficulties in regulating their emotions. These psychological vulnerabilities can contribute to the adoption of binge watching as a maladaptive coping mechanism. The present study aimed to investigate the potential mediating role of vulnerable narcissism and emotion dysregulation in the relationship between CEA and binge watching. Additionally, this study examined whether this model was gender-invariant. A sample of 1082 emerging adults, aged 18 to 25 (50% women), completed self-report questionnaires. The results revealed significant direct and indirect relationships among all the variables of interest, and the model was found to be invariant across genders. Notably, both men and women who experienced CEA and exhibited higher levels of vulnerable narcissism and emotion dysregulation reported engaging in higher levels of binge watching. These findings highlight the importance of the early identification of CEA, the implementation of targeted interventions, and the provision of trauma-informed care to address the negative consequences of CEA and mitigate the development of binge-watching behaviors. Moreover, the results emphasize the need for tailored prevention and intervention programs that address vulnerable narcissism and emotion dysregulation as potential pathways to inform effective therapeutic strategies.

## 1. Introduction

Childhood emotional abuse (CEA) refers to a pervasive type of mistreatment wherein a caregiver intentionally and continuously inflicts emotional harm on a child [1]. This form of abuse encompasses actions like demeaning, mocking, rejecting, or consistently criticizing the child, which creates an environment marked by invalidation. The harmful consequences of CEA on individuals’ psychological well-being have been widely acknowledged, leading to significant repercussions in different areas of their lives [2,3,4]. The above studies have shown that the impacts of CEA extend into adulthood, affecting both personal and professional life, as individuals often struggle with perfectionism, neuroticism, and maladaptive coping mechanisms such as workaholism. Additionally, CEA has been associated with the development of problematic behaviors, including excessive internet use, which may arise from dissociative experiences and a lack of mindfulness. These findings highlight the profound and pervasive effects of CEA, emphasizing the need to understand its role in the formation of psychological vulnerabilities and maladaptive behaviors that persist throughout an individual’s life. One area of growing interest is the relationship between CEA and the development of vulnerable narcissism. Vulnerable narcissism is characterized by a fragile sense of self-worth, heightened sensitivity to criticism, and a persistent need for external validation [5]. Many studies examining the developmental origins of pathological narcissism, such as childhood maltreatment, have predominantly focused on its overt form [6]. Nonetheless, research indicates that vulnerable narcissism can be equally detrimental [7]. This highlights the importance of investigating potential risk factors for vulnerable narcissism in individuals who have experienced childhood maltreatment [6]. The decision to concentrate on vulnerable narcissism in this study is also based on its distinct link to CEA. Evidence suggests that individuals exposed to prolonged invalidation and criticism during childhood are more likely to develop the fragile self-esteem and increased emotional sensitivity associated with vulnerable narcissism [7]. Therefore, the connection between CEA and vulnerable narcissism is arguably more aligned with the emotional damage and persistent invalidation that characterize emotional abuse. Research indicates that CEA is linked to the emergence of vulnerable narcissistic tendencies in adulthood. The chronic invalidation and reproach experienced during childhood can undermine a child’s self-esteem and contribute to the development of a fragile self-image [8]. Consequently, individuals may become highly responsive to criticism and heavily reliant on external validation to uphold their self-worth. Those who have encountered CEA may display various attributes associated with vulnerable narcissism, such as an insatiable need for attention, admiration, and approval from others [9]. They may constantly seek validation and reassurance as a way to counteract the deep-rooted feelings of inadequacy stemming from the emotional abuse endured during their formative years. Furthermore, the emotional abuse experienced in childhood can profoundly influence one’s interpersonal relationships and self-perception [10]. It may lead to a heightened vigilance towards perceived threats of rejection or criticism, resulting in defensive behaviors and difficulties in establishing genuine and trusting connections with others. For the above reasons, individuals with narcissist traits often struggle with difficulties in managing and responding to emotional experiences appropriately; thus, the connection between vulnerable narcissism and emotion dysregulation has become an increasingly intriguing topic in the field of psychology. It appears that the relationship between these two constructs involves a complex interplay involving self-esteem, emotional responses, and strategies for regulation. Hence, the decision to focus on overall emotion regulation difficulties, rather than a specific aspect of emotional regulation, was driven by the aim to have a broader understanding of how individuals with vulnerable narcissistic traits might struggle across various regulatory domains. This holistic approach aligns with the multifaceted emotional difficulties often reported by those with vulnerable narcissism, as they tend to experience intense emotions across different situations and struggle with diverse regulatory strategies [11]. Additionally, the findings from this study could provide a strong foundation for future research to explore more targeted hypotheses, thereby contributing to a deeper understanding of the relevant literature. Present research suggests that individuals displaying vulnerable narcissistic tendencies often encounter intense emotions and possess an elevated sensitivity to situations that could potentially threaten their self-esteem [12]. Consequently, they may encounter difficulties in managing and regulating their emotions, struggling to effectively cope with emotional distress. This may manifest as frequent shifts in mood or intense emotional reactions when they perceive criticism or rejection [11]. Furthermore, individuals with vulnerable narcissism may experience challenges in regulating their emotions due to their fragile self-esteem. They are more susceptible to experiencing profound negative emotions, such as shame or anger, when their sense of self-worth is compromised [13]. Struggles with emotional regulation can hinder their ability to use effective strategies for managing emotions, frequently leading them to rely on impulsive or maladaptive coping methods.

An ever-rising form of maladaptive coping is binge watching, a practice that has become increasingly common with the advent of streaming platforms. Binge watching refers to the practice of consuming numerous episodes or an entire season of a television series in a single sitting [14]. Binge watching has increasingly been recognized as a form of escapism and emotional regulation. Individuals who struggle with intense emotional distress may use binge watching to disengage from their real-world problems by immersing themselves in fictional narratives [15]. This form of media consumption allows them to temporarily avoid confronting painful emotions, creating a sense of emotional relief and control [16]. For individuals with a history of CEA, the practice of binge watching may thus serve as an avoidance mechanism to escape the lingering emotional scars left by their abuse, such as feelings of inadequacy, worthlessness, or rejection [15,16,17,18]. Indeed, binge watching offers viewers the opportunity to engage in immersive entertainment and escapism, becoming deeply engrossed in the storylines and characters [15]. However, excessive and uncontrolled binge watching can have adverse effects on both physical and mental well-being [16] similarly to other technology-related problematic behaviors [17]. Striking a balance between enjoying the entertainment value of binge watching and practicing healthy viewing habits is crucial for overall wellness. Research has indicated that individuals who struggle with regulating their emotions may turn to binge watching as a maladaptive coping mechanism. Specifically, it appears that individuals who face challenges in regulating their emotions are more likely to engage in excessive binge watching as a form of emotional escape or self-soothing [19]. Binge watching offers a temporary escape from emotional distress by acting as both a distraction and a way to manage or dull intense emotions. By immersing themselves in continuous episodes of TV series, individuals can briefly detach from their own emotional experiences and enter a fictional world [15]. This diversion provides a sense of control and a break from real-life pressures. Additionally, binge watching can serve as a method of emotional regulation, delivering comfort and solace. The varied emotional content of TV series, ranging from joy and excitement to sadness, can evoke a wide array of feelings in viewers [19]. For those struggling with emotion dysregulation, this emotional engagement may help them modulate or manage their own emotional states. Binge watching thus offers a consistent and controlled emotional experience, allowing individuals to adjust their mood and find temporary relief from emotional distress [20]. For those with vulnerable narcissistic tendencies, who are especially prone to intense emotional fluctuations, binge watching can provide a temporary sense of relief and emotional regulation. In fact, this behavior mirrors other impulsive coping mechanisms seen in vulnerable narcissism, potentially reinforcing the role of emotional dysregulation as a key factor linking vulnerable narcissism to binge-watching behavior [6,7]. Vulnerable narcissists may struggle with interpersonal relationships and experience social isolation. Binge watching can thus offer a sense of companionship through engagement with characters and storylines, providing a substitute for real social interactions and helping to alleviate feelings of loneliness [6,7].

The literature has been trying to address the question of whether gender has any role in binge-watching behaviors. Numerous studies have been conducted, yielding mixed and inconclusive findings. Steins-Loeber et al. [21], for instance, discovered no noteworthy gender distinctions in binge-watching tendencies. However, contrasting findings have been presented by Starosta et al. [22], who suggest that women may exhibit stronger motivation for entertainment, coping with feelings of loneliness, and seeking social connection through binge watching compared to men. Furthermore, in regards to maladaptive motives behind binge watching, Alfonsi et al. [19] found that men tend to score higher on coping/escapism dimensions. This body of research highlights the need for further investigation to fully comprehend the potential role of gender in binge-watching behaviors.

Examining the association between CEA, vulnerable narcissism, emotion dysregulation, and binge watching contributes to our theoretical comprehension of how early negative experiences can impact the formation of ineffective coping mechanisms. This investigation provides valuable insights into the psychological processes and mechanisms that may underlie the link between CEA and subsequent engagement in binge-watching behaviors. CEA is widely acknowledged for its adverse effects on individuals’ psychological well-being, emphasizing the importance of understanding how it contributes to the development of specific psychological vulnerabilities and coping patterns. This understanding sheds light on the enduring consequences of CEA on individuals’ emotional and behavioral functioning. An exploration of the role played by vulnerable narcissism and emotion dysregulation aids in identifying specific psychological factors that may be involved in the emergence and maintenance of counterproductive coping strategies, such as binge watching. Grasping the intricate interplay among CEA, vulnerable narcissism, emotion dysregulation, and binge watching can inform the assessment, diagnosis, and treatment methods for individuals who employ binge-watching behaviors. It can guide the development of targeted interventions that address the underlying vulnerabilities and encourage the adoption of healthier coping mechanisms, strategies for emotional regulation, and habits related to media consumption. 

**Figure 1:**
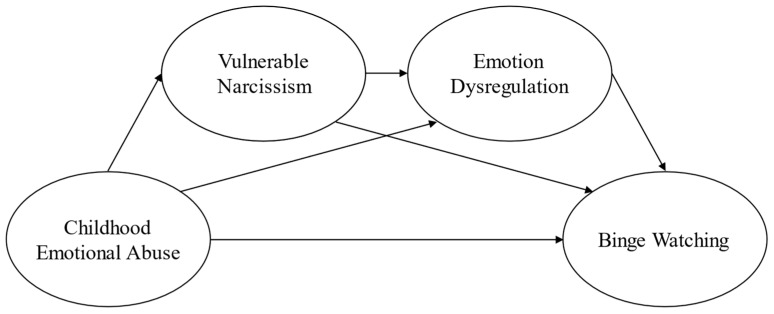
Hypothesized model.

Building upon the aforementioned considerations, the present investigation aims to address the existing research gaps. The primary objective of this study is to examine if vulnerable narcissism and emotion dysregulation sequentially mediate the relationship between CEA and binge watching (Figure 1). In an explorative fashion, the current study also verified if the hypothesized model was invariant across genders.

## 2. Materials and Methods

### 2.1. Participants

**Figure 2:**
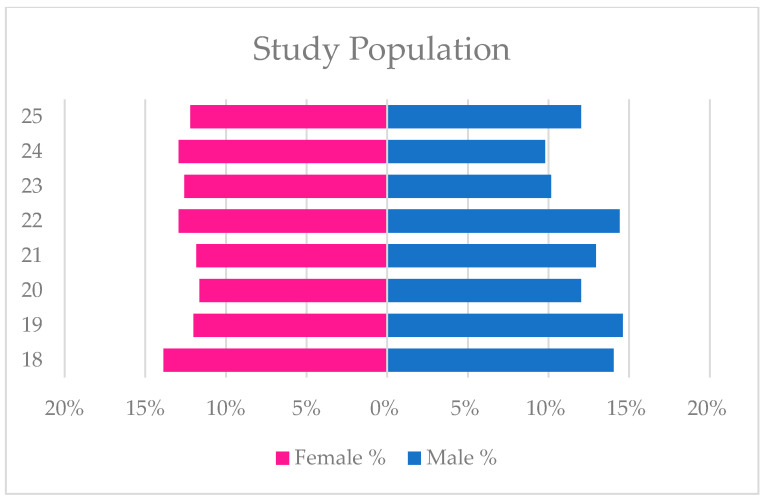
Study population.

The present study comprised a sample of 1082 young adults residing in Italy, with an equal gender distribution of 541 females and 541 males (see Figure 2). The participants’ ages ranged from 18 to 25 years (M = 21.39, SD = 2.31). 

Recruitment of participants took place online. Although this approach might have introduced some bias, online recruitment was chosen for several reasons, each contributing to the overall validity and representativeness of the sample. Firstly, online recruitment allowed us to reach participants from various regions across Italy, rather than being restricted to a specific city or locality. Secondly, since individuals who engage in binge watching are likely to be active internet users, online platforms provided an appropriate medium for reaching this demographic. Thirdly, online recruitment offers convenience and accessibility for participants, which may have increased participation rates. Finally, utilizing a range of online platforms enabled us to engage a diverse group of participants. To ensure a balanced and diverse sample, we recruited participants through various online channels. These included social media platforms (e.g., Facebook), online forums (e.g., Reddit), mailing lists (e.g., university email lists), online community groups (e.g., LinkedIn Groups), popular blogs and websites (e.g., those with significant followings), and online TV series communities (e.g., Netflix fan communities).

The inclusion criteria required individuals to be between 18 and 25 years old, be fluent in Italian, have watched media content in one sitting within the past six months, and spend at least 7 h per week watching media content. The choice to focus on young adults was made to provide a clearer picture of binge-watching behaviors in a demographic where these behaviors are pronounced and potentially the most impactful, facilitating more targeted and relevant research outcomes [18,22].

A Monte Carlo power analysis for mediation models indicated that a minimum of 1030 participants would be necessary to reach a statistical power of 0.80 [23].

With regards to educational background, 16% of the participants had completed middle school, 51% held a high school diploma, 30% had obtained a university degree, and 3% had pursued postgraduate studies. Regarding occupational status, 47% of the participants were students, 11% were unemployed, 31% were employed, and 11% were self-employed. Marital status-wise, 40% of the participants were single, 34% were engaged, 16% were cohabiting, and 10% were married. To ensure the representativeness of our sample, we compared its characteristics with those reported in similar Italian studies. Our sample’s demographic and behavioral profiles were found to be consistent with those of participants in comparable research (e.g., [3,17,18,24,25]). This alignment suggests that our sample adequately reflects the target population, providing higher confidence in the generalizability of our findings.

### 2.2. Procedures

The present study adhered to the ethical principles outlined in the Helsinki Declaration and the Italian Association of Psychology (AIP). Approval for this study was obtained from the Institutional Review Board of the Institute for the Study of Psychotherapy, School of Specialization in Brief Psychotherapies with a Strategic Approach (reference number: ISP-IRB-2023-4). Participants were invited to participate in a thorough online survey, and their completion of the survey was obligatory to ensure the collection of comprehensive data. To prevent any missing responses, we set all survey questions on Google Forms as mandatory, ensuring that participants could not unintentionally leave any answers blank. Only individuals who provided informed consent were included in this study, and their participation was voluntary, without any form of compensation provided. Maintaining the privacy and confidentiality of the participants was of utmost importance throughout all phases of this research.

### 2.3. Measures

#### 2.3.1. Childhood Emotional Abuse

CEA was evaluated using the CEA subscale of the Childhood Trauma Questionnaire—Short Form (CTQ-SF) [26]. The CTQ-SF is a widely used instrument for measuring various forms of childhood trauma, including emotional abuse. In this study, we used the validated Italian version of the instrument [27]. Participants were asked to assess the severity of CEA experienced during their childhood by responding to five items (e.g., “People in my family said hurtful or insulting things to me”). Each item was rated on a 5-point Likert scale, ranging from 1 (never true) to 5 (very often true). The scores from the five items were averaged, with higher scores indicating greater levels of CEA. Previous studies conducted in Italy have established the reliability and validity of the CTQ-SF [3,4]. In this study, Cronbach’s alpha was 0.87.

#### 2.3.2. Vulnerable Narcissism

To measure vulnerable narcissism, the Hypersensitive Narcissism Scale (HSNS) [28] was employed. The HSNS is a well-established self-report instrument designed to measure traits associated with vulnerable narcissism. Specifically, we used the validated Italian version of the instrument [29]. The HSNS is a self-report questionnaire consisting of 10 items that assess various traits associated with vulnerable narcissism. Sample items include “I often interpret the remarks of others in a personal way”. Participants indicate their agreement with each item using a 5-point Likert scale, ranging from 1 (strongly disagree) to 5 (strongly agree). Higher scores on the HSNS indicate greater levels of vulnerable narcissism. Previous studies conducted in Italy have established the reliability and validity of the HSNS [24,29]. In this study, Cronbach’s alpha was 0.80.

#### 2.3.3. Emotion Dysregulation

To assess emotion dysregulation, the Difficulties in Emotion Regulation Scale (DERS-20) [30,31] was utilized. The DERS-20 is a widely recognized self-report questionnaire specifically designed to evaluate the difficulties individuals experience in managing their emotions. In this study, we used the validated Italian version of the instrument [31]. The DERS-20 is a self-report questionnaire comprising 20 items designed to measure difficulties individuals face in regulating their emotions. Examples of items include “When I’m upset, I feel guilty for feeling that way”. Participants are asked to rate each item on a 5-point Likert scale, ranging from 1 (almost never) to 5 (almost always). Higher scores on the DERS-20 indicate higher levels of emotion dysregulation. Previous studies conducted in Italy have established the reliability and validity of the DERS-20 [24,31]. In this study, Cronbach’s alpha was 0.90.

#### 2.3.4. Binge Watching

The assessment of binge watching involved the utilization of the Italian version of the Binge Watching Addiction Questionnaire (BWAQ) [32]. This instrument is designed to evaluate the severity of binge-watching habits among individuals. Specifically, participants were presented with 20 items aimed at gauging the extent of their engagement in binge-watching behaviors. For instance, individuals were asked to indicate the frequency of statements such as “Do you happen to find yourself saying “one more episode and I’ll turn it off” when you watch a TV series?”. Responses to each item were rated on a 5-point Likert scale, ranging from 0 (never) to 4 (always). Average scores were calculated across all 20 items, with higher scores indicating a greater propensity for binge-watching behaviors. Previous studies conducted in Italy have established the reliability and validity of the BWAQ [32,33]. In this study, Cronbach’s alpha was 0.95.

### 2.4. Statistical Analyses

The descriptive statistics and correlations were conducted using IBM SPSS 27 software. The main analyses were conducted utilizing the lavaan package in RStudio.

To investigate the mediation model, a latent variable structural equation modeling (SEM) approach was utilized. The model included CEA as the predictor, vulnerable narcissism as the first mediator, emotion dysregulation as the second mediator, and binge watching as the outcome. A parceling technique was employed to derive the indicators for the latent variables in our model. In this approach, items from the questionnaire were combined into three indicators for each latent variable. This method is considered more effective for model evaluation than using models based solely on individual observed variables (e.g., [34]). The significance of the indirect effects was assessed using the bootstrap-generated bias-corrected confidence interval method with 5000 resamples. The mediation analysis accounted for gender by including it as a predictor for all study variables.

To examine potential distinctions in the structural pathways between boys and girls, we conducted a multigroup path analysis (MGPA) on the proposed model. This analysis aimed to assess structural invariance, which evaluates whether the pattern of relationships among variables is consistent across different groups.

## 3. Results

### 3.1. Descriptive Statistics and Correlations

Table 1 displays the descriptive statistics and correlations among the variables investigated in this study. Key results include significant positive correlations between CEA and vulnerable narcissism (r = 0.39, *p* < 0.05), as well as between CEA and emotion dysregulation (r = 0.31, *p* < 0.05). Vulnerable narcissism also showed a strong positive correlation with emotion dysregulation (r = 0.50, *p* < 0.05). Additionally, binge watching was positively correlated with CEA (r = 0.34, *p* < 0.05), vulnerable narcissism (r = 0.30, *p* < 0.05), and emotion dysregulation (r = 0.35, *p* < 0.05). All the correlations are thus significant and moderate in strength. These findings suggest that higher levels of emotional abuse, narcissism, and dysregulation are associated with increased binge watching.

### 3.2. Mediation Model

**Figure 3:**
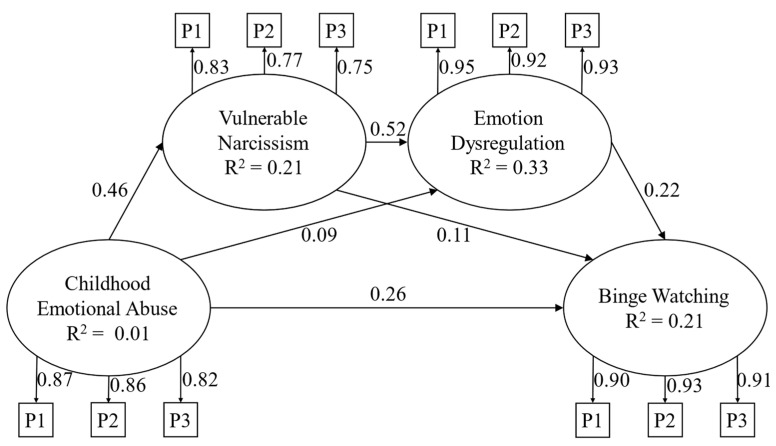
Structural model. Note: *p* = parcel; only the path coefficients of the direct effects are reported for presentation and clarity purposes; paths from gender were not presented for presentation and clarity purposes.

The proposed model was evaluated through structural equation modeling (SEM) incorporating latent variables (Figure 3). The results revealed a satisfactory fit between the model and the data: χ2(56) = 121.11, *p* < 0.001, CFI = 0.99, RMSEA = 0.03 (90% CI = 0.03−0.04), and SRMR = 0.02. The effect sizes in our study reveal varying degrees of practical significance across different relationships (see Table 2). The strongest effects are observed between CEA and all the other study variables, between vulnerable narcissism and emotion dysregulation, and between emotion dysregulation and binge watching. Importantly, while some effect sizes are smaller, significant direct and indirect paths were observed among all the variables analyzed, thus still providing valuable insights into the complex interplay between these variables. 

### 3.3. Moderating Role of Gender

To examine potential distinctions in the structural pathways between boys and girls, a multigroup path analysis (MGPA) was conducted on the proposed model. An analysis was performed by comparing a constrained model, where the paths in the hypothesized model were set as equal for both groups, χ2(102) = 177.27, *p* < 0.001, and CFI = 0.99, with an unconstrained model, allowing all paths to vary across the two groups, χ2(96) = 166.07, *p* < 0.001, and CFI = 0.99. The fit indices of the unconstrained model did not display significant deviation from the constrained model, indicating structural equivalence between the two groups, Δχ2(6) = 10.77, *p* = 0.10, and ΔCFI = 0.001. Consequently, the relationships were found to be similar and consistent for both boys and girls. 

## 4. Discussion

The primary objective of this research was to examine if vulnerable narcissism and emotion dysregulation act as mediators in the link between CEA and binge watching. This study’s outcomes reveal that both vulnerable narcissism and emotion dysregulation play a mediating role in the aforementioned relationship. These findings have noteworthy implications for advancing our comprehension of the underlying processes involved in the development of binge-watching behaviors in individuals. CEA and vulnerable narcissism share interconnected aspects that contribute to their association. When a child endures emotional abuse during their formative years, their self-esteem and self-worth can be severely undermined [8]. The constant barrage of criticism, rejection, and belittlement can cause them to internalize these negative messages, resulting in a pervasive sense of inadequacy that aligns with the vulnerability characteristic of vulnerable narcissism. Moreover, to cope with the pain and humiliation inflicted upon them, children may develop defensive mechanisms, including the adoption of narcissistic features [9]. By adopting a self-centered worldview and seeking external validation, they aim to protect themselves from further emotional harm, which may manifest as vulnerable narcissism later in life. Furthermore, the experience of emotional abuse can profoundly affect an individual’s ability to handle criticism [5]. Those who have endured emotional abuse may become hypersensitive to any form of criticism or rejection, which resonates with the central trait of vulnerable narcissism, wherein individuals overreact to perceived slights or criticism. Lastly, some individuals who have experienced CEA may internalize the abusive behaviors and beliefs imposed upon them [9,10]. Consequently, they may externalize these patterns of abuse in their adult relationships, exhibiting narcissistic behaviors such as a need for admiration, manipulation, and a lack of empathy, characteristics commonly associated with vulnerable narcissism [5,9]. Vulnerable narcissism and emotion dysregulation are closely intertwined and influence each other in various ways. Individuals with vulnerable narcissism typically possess a fragile sense of self-esteem, which makes them highly sensitive to perceived criticism or slights [12]. Consequently, they tend to react emotionally and feel vulnerable when their self-esteem is challenged or when feelings of shame and inadequacy arise. Additionally, vulnerable narcissists are prone to being hypersensitive to rejection and abandonment, often misinterpreting neutral or ambiguous cues as signs of rejection [11]. As a result, they may experience intense emotional reactions like sadness, anger, or anxiety. This hypersensitivity to rejection can create difficulties in effectively regulating their emotions, as they are more likely to have heightened emotional responses to threats against their self-worth [12]. Moreover, vulnerable narcissists tend to engage in dichotomous thinking, viewing themselves as either completely superior or deeply flawed without recognizing the shades of gray in between [35]. This rigid thinking pattern impairs their ability to tolerate and regulate complex or conflicting emotions since they struggle to find a middle ground between extremely positive and negative self-evaluations. Furthermore, individuals with vulnerable narcissism may struggle with internalizing and processing their emotions appropriately, resorting instead to externalizing their emotions through blame, attention-seeking, or manipulative behaviors [11,13]. This externalization of emotions represents an effective strategy for regulating and expressing emotions, as it fails to address the underlying emotional experiences in a healthy or constructive manner [18,24]. Lastly, vulnerable narcissists often experience intense emotional fluctuations, characterized by significant highs and lows [12]. Maintaining emotional stability becomes challenging for them, resulting in rapid mood shifts or affective changes. These emotional fluctuations pose obstacles to effectively regulating their emotional experiences, potentially leading to impulsive behaviors, emotional outbursts, and difficulties in finding adaptive coping strategies. Given the aforementioned points, it is plausible to posit that vulnerable narcissism acts as a mediating factor between CEA and emotion dysregulation. More specifically, vulnerable narcissism can serve as a mediator in this relationship by impacting crucial elements such as self-esteem, emotional processing, and coping mechanisms. Furthermore, for individuals with vulnerable narcissistic traits, who frequently experience profound emotional swings, binge watching can serve as a temporary escape and a means of emotional regulation. This behavior parallels other impulsive coping strategies often observed in vulnerable narcissism, suggesting that emotional dysregulation may play a crucial role in connecting vulnerable narcissism with binge-watching tendencies [6,7]. People with vulnerable narcissism often face challenges in their interpersonal relationships and may experience social isolation. Engaging in binge watching can create a sense of companionship through the connection with characters and narratives, offering a surrogate for genuine social interactions and helping to mitigate feelings of loneliness [6,7]. This temporary immersion in media content can provide emotional comfort and a sense of belonging, further reinforcing the link between emotional dysregulation and binge-watching behaviors in vulnerable narcissists. The relationship between emotion dysregulation and binge watching is a recent area of investigation. There are several ways in which these two factors can be connected. Individuals with emotion dysregulation often struggle with effectively managing their emotional states, leading them to seek out strategies for emotional relief [19]. Binge watching can thus serve as a coping mechanism for these individuals by providing an escape from emotional distress. Immersing oneself in a fictional world allows for temporary distraction from real-life challenges, offering a form of emotional numbing that can provide momentary relief from overwhelming feelings [15]. This act of binge watching provides a form of emotional numbing, offering momentary relief from overwhelming emotions. This aligns with research suggesting that media consumption can act as an emotional refuge, helping individuals to avoid confronting their internal struggles [20,36]. Furthermore, binge watching can function as a form of emotion regulation. Different types of media content evoke specific emotional responses, and individuals may engage in binge watching to either elevate their mood or process their emotions. For example, someone experiencing sadness might turn to a comedy series to improve their mood or find comfort in relatable characters [20,36]. This behavior is consistent with findings that suggest individuals use media as a tool for managing their emotional states and coping with feelings of loneliness or distress [37]. Additionally, the deep emotional engagement with TV series and films can create a heightened sense of connection, which may be particularly appealing to individuals with emotion dysregulation. These individuals may find it easier to navigate their emotions through the narratives and characters on screen rather than directly confronting their own emotional experiences [37]. Moreover, emotion dysregulation is frequently linked to impulsive behaviors [20,21]. Impulsive binge watching can act as a form of self-soothing or self-gratification, offering immediate pleasure and distraction from emotional discomfort. This impulsive engagement with media content can provide temporary relief but may also reinforce maladaptive coping strategies [20,21]. Considering these insights, it is plausible that emotion dysregulation mediates the relationship between vulnerable narcissism and binge watching. Emotion dysregulation may facilitate emotional escape, mood management, avoidance of self-reflection, and the pursuit of gratification in the context of vulnerable narcissism.

The second objective of this study was to examine the consistency of the proposed model across genders. The results indicated that the structural relationships within the model were similar for both men and women, with no significant differences observed. This suggests that the mediating roles of vulnerable narcissism and emotion dysregulation in the relationship between CEA and binge watching are equally relevant for both genders. These findings are important as they highlight the generalizability of the model, suggesting that these psychological processes operate similarly across genders despite potential societal or cultural expectations about emotional regulation and media consumption. Although previous research has identified gender differences in psychological factors related to binge watching, such as motivations and emotional responses [19,21,22], the current study’s findings suggest that the key factors of CEA, vulnerable narcissism, and emotion dysregulation may exert comparable influences on binge-watching behavior across men and women. This result could imply that gender-specific differences often reported in other studies might be more closely tied to socialization processes or external gender norms, rather than the internal psychological mechanisms examined here. Further research could explore whether additional gender-related factors, such as emotional expressivity, coping styles, or cultural expectations, might still influence these relationships in ways that were not captured in the current model. By considering these broader gender dynamics, this study underscores the importance of addressing both shared and gender-specific factors in understanding how CEA and related vulnerabilities contribute to binge-watching behaviors.

## 5. Conclusions

### 5.1. Limitations

There are several limitations in our research that should be addressed. Firstly, the cross-sectional design used in our study restricts our ability to establish causal relationships between the variables examined. To better understand the long-term effects of CEA, vulnerable narcissism, and emotion dysregulation on binge watching, future research should utilize longitudinal designs that track participants over time, allowing for the observation of changes in behavior and emotional regulation across different stages of life. In addition, our reliance on self-reported data introduces the possibility of interpretive bias, as participants’ subjective perceptions could influence their responses, and it is also important to note that Likert scales may not fully capture the nuanced experiences associated with these constructs. To mitigate these biases, future studies could incorporate deeper (e.g., longer self-report instruments with several subscales) and/or objective measures such as physiological assessments (e.g., heart rate variability, cortisol levels) to measure emotional stress and dysregulation more accurately. Moreover, behavioral data from streaming platforms, such as actual viewing patterns and time spent binge watching, could provide more reliable insights into binge-watching behaviors, reducing the reliance on self-reports. Furthermore, this study did not account for potential confounding variables such as histories of other types of abuse (e.g., physical or sexual abuse) or pre-existing psychological conditions. These factors may influence the observed relationships, and future research should include them to provide a more comprehensive understanding of how multiple adversities interact with CEA, vulnerable narcissism, and emotion dysregulation in contributing to binge-watching behaviors. In addition, selection bias due to the voluntary nature of participation in online surveys is a potential limitation. Future studies should aim for a more diverse participant pool by employing random sampling methods or combining online and offline recruitment strategies. Moreover, as our data collection was exclusively online, individuals without internet access or those less inclined to participate in online surveys may be underrepresented. Future research should consider incorporating in-person interviews or offline data sources to ensure a more representative sample. Furthermore, another limitation of this study is the cultural specificity of the sample, which included only young Italian adults. Cultural factors, such as media consumption habits and attitudes toward emotional expression, may influence binge-watching behaviors, narcissism, and emotional regulation. This limits the generalizability of our findings to other populations. Future research should explore these relationships across different cultural settings to determine if the observed patterns hold true in more diverse contexts. Finally, another limitation of this study is the lack of direct measurement of socioeconomic status (SES). Although we included related indicators such as the educational level and occupational status, future research should consider incorporating more precise measures of SES to better understand its role in the study variables.

### 5.2. Future Implications

The findings of this study provide valuable insights into the intricate connections between CEA, vulnerable narcissism, emotion dysregulation, and binge watching. While these results are significant for understanding these connections within our sample of young adults in Italy, caution should be exercised when generalizing these findings to other populations. Nonetheless, this knowledge is of great importance for clinical practitioners as it can guide them in developing effective interventions and strategies to address these issues.

The findings of this study have significant clinical implications, offering guidance for clinicians working with individuals who engage in binge watching and have a history of CEA. Clinicians could use these findings to identify and address symptoms of vulnerable narcissism and emotional dysregulation by incorporating targeted assessments. For instance, specific diagnostic tools and questionnaires such as the ones used in this study might be utilized to evaluate the presence and severity of vulnerable narcissism and emotion dysregulation, which could help in developing individualized treatment plans. Since CEA is a traumatic experience with long-lasting effects, trauma-informed therapy could be important. Clinicians might consider strategies such as cognitive–behavioral therapy (CBT) and eye movement desensitization and reprocessing (EMDR) to help clients process and heal from their trauma. To address emotion dysregulation, clinicians could teach practical emotion regulation skills through techniques like mindfulness, emotion-focused therapy, and distress tolerance strategies. These approaches might help clients recognize, tolerate, and manage their emotions more effectively. In managing vulnerable narcissism, clinicians could focus on interventions that aim to improve self-esteem and resilience. Techniques such as schema therapy and self-compassion training might be useful in addressing the underlying vulnerabilities associated with this trait. Additionally, clinicians could educate individuals, families, and communities about the links between CEA, vulnerable narcissism, emotion dysregulation, and binge watching. Early intervention and prevention strategies might emphasize supporting CEA victims and promoting healthy coping mechanisms from a young age.

To strengthen the validity and scope of our understanding, future research should aim to replicate and build upon these findings across a range of diverse populations. This should involve examining different age groups, cultural contexts, and clinical populations. Such efforts will help determine whether the observed relationships are generalizable and provide a more thorough insight into how childhood emotional abuse (CEA) and related factors affect individuals in various settings. Additionally, future research endeavors can focus on evaluating the effectiveness of interventions that specifically target vulnerable narcissism, emotion dysregulation, and binge watching in individuals with a history of CEA. Through such investigations, we can discern which interventions are most successful in addressing the underlying factors and reducing binge-watching behaviors. The insights gained from these studies have the potential to significantly influence clinical practice and the development of treatment guidelines. 

## Figures and Tables

**Table 1:** Descriptive analyses and correlations.

Variables	M	SD	Ske	Kur	α	1	2	3
1. Childhood Emotional Abuse	1.99	1.01	0.95	−0.03	0.87	-	-	-
2. Vulnerable Narcissism	2.86	0.71	−0.16	−0.10	0.80	0.39 *	-	-
3. Emotion Dysregulation	2.59	0.70	0.15	−0.49	0.90	0.31 *	0.50 *	-
4. Binge Watching	2.22	0.81	0.41	−0.44	0.95	0.34 *	0.30 *	0.35 *

Note: N = 1082; * *p* < 0.01.

**Table 2:** Path Estimates, SEs, and 95% CIs.

Variable	*β*	*p*	SE	CI	CI
				LL	UL
Direct Effect					
Childhood Emotional Abuse → Vulnerable Narcissism	0.46	<0.001	0.02	0.26	0.35
Childhood Emotional Abuse → Emotion Dysregulation	0.09	0.01	0.03	0.02	0.13
Childhood Emotional Abuse → Binge Watching	0.26	<0.001	0.03	0.15	0.27
Vulnerable Narcissism → Emotion Dysregulation	0.52	<0.001	0.04	0.54	0.71
Vulnerable Narcissism → Binge Watching	0.11	0.01	0.06	0.03	0.24
Emotion Dysregulation → Binge Watching	0.22	<0.001	0.05	0.14	0.32
Indirect Effect via Vulnerable Narcissism					
Childhood Emotional Abuse → Emotion Dysregulation	0.24	<0.001	0.02	0.15	0.23
Childhood Emotional Abuse → Binge Watching	0.05	0.02	0.02	0.01	0.08
Indirect Effect via Emotion Dysregulation					
Childhood Emotional Abuse → Binge Watching	0.02	0.02	0.01	0.004	0.03
Vulnerable Narcissism → Binge Watching	0.12	<0.001	0.03	0.09	0.21

Note: *p*—level of significance; SE—standard error; CI—confidence interval; LL—lower limit; UL—upper limit.

## Data Availability

The data presented in this study are available on request from the corresponding author (the data are not publicly available due to privacy and ethical restrictions).
